# Potential Use of Antimicrobial Peptides as Vaginal Spermicides/Microbicides

**DOI:** 10.3390/ph9010013

**Published:** 2016-03-11

**Authors:** Nongnuj Tanphaichitr, Nopparat Srakaew, Rhea Alonzi, Wongsakorn Kiattiburut, Kessiri Kongmanas, Ruina Zhi, Weihua Li, Mark Baker, Guanshun Wang, Duane Hickling

**Affiliations:** 1 Chronic Disease Program, Ottawa Hospital Research Institute, Ottawa, Ontario K1H 8L6, ON, Canada; fscinrsr@ku.ac.th (N.S.); ralonzi@ohri.ca (R.A.); wkiattiburut@ohri.ca (W.K.); kessiri.kon@mahidol.ac.th (K.K.); ruina.zhi@gmail.com (R.Z.); dhickling@toh.ca (D.H.); 2 Department of Obstetrics and Gynecology, Faculty of Medicine, University of Ottawa, Ottawa, Ontario K1H 8L6, ON, Canada; 3 Department of Biochemistry, Microbiology, Immunology, Faculty of Medicine, University of Ottawa, Ottawa, Ontario K1H 8M5, ON, Canada; 4 Department of Zoology, Faculty of Science, Kasetsart University, Bangkok 10900, Thailand; 5 Division of Dengue Hemorrhagic Fever Research Unit, Office of Research and Development, Faculty of Medicine Siriraj Hospital, Mahidol University, Bangkok 10700, Thailand; 6 Key Laboratory of Reproduction Regulation of NPFPC, Shanghai Institute of Planned Parenthood Research, and School of Public Health, Fudan University, Shanghai 200032, China; iamliweihua@foxmail.com; 7 Reproductive Proteomics, Department of Science and Information technology, University of Newcastle, Callaghan Drive, Newcastle, NSW 2308 Australia; mark.baker@newcastle.edu.au; 8 Department of Pathology and Microbiology, College of Medicine, University of Nebraska Medical Center, 986495 Nebraska Medical Center, Omaha, NE 68198-6495, USA; gwang@unmc.edu; 9 Division of Urology, Department of Surgery, Faculty of Medicine, University of Ottawa, Ottawa, Ontario K1Y 4E9, ON, Canada

**Keywords:** antimicrobial peptide, spermicide, spermicidal antimicrobial peptide, vaginal microbicide, vaginal contraceptive, sexually transmitted infection, vaginitis, LL-37, cathelicidin, hCAP-18

## Abstract

The concurrent increases in global population and sexually transmitted infection (STI) demand a search for agents with dual spermicidal and microbicidal properties for topical vaginal application. Previous attempts to develop the surfactant spermicide, nonoxynol-9 (N-9), into a vaginal microbicide were unsuccessful largely due to its inefficiency to kill microbes. Furthermore, N-9 causes damage to the vaginal epithelium, thus accelerating microbes to enter the women’s body. For this reason, antimicrobial peptides (AMPs), naturally secreted by all forms of life as part of innate immunity, deserve evaluation for their potential spermicidal effects. To date, twelve spermicidal AMPs have been described including LL-37, magainin 2 and nisin A. Human cathelicidin LL-37 is the most promising spermicidal AMP to be further developed for vaginal use for the following reasons. First, it is a human AMP naturally produced in the vagina after intercourse. Second, LL-37 exerts microbicidal effects to numerous microbes including those that cause STI. Third, its cytotoxicity is selective to sperm and not to the female reproductive tract. Furthermore, the spermicidal effects of LL-37 have been demonstrated *in vivo* in mice. Therefore, the availability of LL-37 as a vaginal spermicide/microbicide will empower women for self-protection against unwanted pregnancies and STI.

## 1. The Need for New Types of Contraceptives

As the world population continues to grow at an exponential rate, so does the need for novel contraceptives. Oral contraceptive pills (OC), formulated with a balanced combination of estrogen and progestin, are praised for their highest contraceptive efficacy and reversibility. The high, non-physiological dosages of estrogen and progestin in OCs induce an imbalance of reproductive hormones in the hypothalamus-pituitary-ovary axis, and subsequently a failure in ovulation. Progestin also thickens the cervical mucus, thus impeding sperm from swimming into the uterus and oviduct. These features empower women to protect themselves from unwanted pregnancies and therefore have become the most widely used form of contraception. However, these sex steroid based pills are not without risks, as estrogen and progestin are also involved in other physiological processes outside of ovulation and pregnancy. Excess circulating amounts of either hormone can lead to undesirable side effects. The most common minor side effects include nausea, weight gain and acne formation, whereas major side effects can include thromboembolism, hypertension, hyperlipidemia, cardiovascular disease, and breast and cervical malignancies. Long-term exposure to these hormones has also been shown to cause vaginal atrophy, which can lead to local symptoms and predisposition to infection. Women with a personal or family history of the aforementioned medical abnormalities or those who engage in high-risk behaviours such as smoking are medically advised against the use of OCs [1]. Estrogen and/or progestin can also be systemically administered via transdermal patch, subdermal implant, intradermal injection, vaginal ring, or intrauterine device (IUD). Regardless of the administration route, the side effects associated with estrogen/progestin are similar. Recent studies further suggest that the intradermally injected progestin, depo-medroxyprogesterone acetate (DMPA), may induce the thinning of the vaginal epithelium, and therefore increase the infection risk of microbes [2,3]. What is more, there is now growing concern about bioaccumulation of these hormonally based contraceptives and their associated negative impact on ecologic systems of all scales [4,5]. Therefore, development of reversible, locally acting and non-hormonal contraceptives should be considered.

As the global population increases, so does the rate of sexually transmitted infection (STI). HIV/AIDS continues to be a prevalent condition, affecting approximately 37 million people worldwide (http://www.who.int/hiv/data/epi_core_july2015.png?ua=1). The vaginal mucosa is the portal by which HIV from semen of seropositive men infects women following intercourse [6]. Many other viruses, bacteria, yeasts and protozoa also cause sexually transmitted infection (Table 1) via this route. Physical barriers such as male and female condoms prevent transmission of potential pathogens between both sexes. STI-induced microorganisms, as well as sperm, cannot penetrate through the condom layer, made from polyurethane or polyisoprene [1]. Therefore, the use of condoms is considered part of a safe sex practice for both men and women, as they are protected against unwanted pregnancies and STIs. However, the main drawback of condom use is decreased sensation and sensual pleasure during intercourse. Also, improper use and breakage during intercourse are concerns of the contraceptive efficacy of condoms [1]. Development for chemical spermicides and microbicides for vaginal use is therefore much needed.

## 2. Vaginally Administered Compounds with Dual Actions as Spermicides and Microbicides

Clearly, compounds with dual spermicide/microbicide action need to be developed. Various approaches to this can be considered, including microbicide screening against compounds with known spermicidal activity. Indeed, this was the approach taken for the spermicide, nonoxynol-9 (N-9). However, the final development of N-9 into a vaginal microbicide was not successful due to its deleterious effects on the vaginal epithelium as well as its microbicide inefficacy. Apparently, the development of N-9 into a spermicide over 50 years ago did not take into account the physiology of sperm function, the knowledge of which has been slowly unfolding over the past decades. Details of the current knowledge on sperm physiology/biochemistry are therefore given herein for consideration of the development of the next generation of vaginal contraceptive/microbicide compounds.

### 2.1. Mechanisms on How Mammalian Sperm Gain Fertilizing Ability

The fertilizing potential of sperm in the ejaculate correlates with a number of basic parameters including motility, concentration, total sperm number, and sperm morphology [7]. However, sperm can fertilize eggs only after they undergo the capacitation process. This occurs naturally in the female reproductive tract, and can be mimicked *in vitro* by incubating isolated sperm relatively free of seminal plasma in a medium containing calcium, bicarbonate and albumin. “Capacitation” defines the overall biochemical and physiological changes that allow sperm to bind to the egg and then enter into its cytoplasm. During capacitation significant changes occur on the sperm plasma membrane [8]. This is partly attributed to a cholesterol efflux [8,9], which subsequently increases overall sperm plasma membrane fluidity, preparing sperm for the two membrane fusion events essential for completing the fertilization process. The first event is part of the onset of the acrosome reaction. The acrosome is a membrane enveloped cap-like structure that is underneath the plasma membrane of the sperm head anterior. Upon exposure to stimulators such as zona pellucida (ZP) glycoproteins and progesterone, calcium is rapidly transported into sperm, and the acrosome reaction is initiated with the multi-site fusion between the sperm anterior head plasma membrane and the outer acrosomal membrane. This membrane fusion results in the pore formation in the sperm head anterior and finally exocytosis of the acrosomal content, mainly composed of hydrolytic enzymes, into the surrounding. These hydrolytic enzymes digest the egg vestments (networks of cumulus cell layers composed of proteo-glycosaminoglycans and the egg extracellular matrix-the ZP), thus facilitating sperm to swim towards the egg plasma membrane [8]. Without the completion of the acrosome reaction, fertilization cannot take place [8,10]. On the other hand, if the acrosome reaction is completed prematurely, sperm will have problems penetrating the egg vestments and also binding to the egg ZP.

Once acrosome reacted sperm penetrate through the ZP, they reach and bind to the egg plasma membrane. At this time the second membrane fusion event occurs between the plasma membrane of the head (post-acrosomal) region of an acrosome reacted sperm and the egg plasma membrane. This fusion is immediately followed by incorporation of the whole sperm into the egg proper and this signifies that fertilization has occurred.

The increase of the membrane fluidity due to cholesterol efflux also leads to a change in the sperm movement patterns. Sperm swim with a progressive forward pattern before capacitation. Capacitated sperm, however, swim with “hyperactivated motility” patterns, which are whiplash like with a high amplitude of lateral head (ALH) displacement. These swimming patterns endow sperm with a high thrusting force, facilitating them to penetrate through the egg vestments [8,11]. CatSper calcium cation channels play an integral role in sperm acquisition of hyperactivated motility patterns [12]. Male mice genetically deleted of *CatSperδ* are infertile; despite normal sperm production, sperm of these knockout mice cannot move with hyperactivated motility patterns [13]. Changes in the sperm plasma membrane composition during capacitation also lead to the exposure of sperm head surface molecules that are responsible for binding to the egg ZP in a species specific manner [8]. These ZP binding molecules are localized to the sperm anterior head plasma membrane overlying the acrosome. To date, more than 15 proteins, as well as a male germ cell specific sulfoglycolipid, sulfogalactosylglycerolipid (SGG, aka seminolipid) [14,15,16], have been shown for their affinity for the egg ZP. Results from knockout mouse studies indicate that most of these proteins and SGG are not essential for sperm fertilizing ability, since the knockout male mice remain fertile [17]. These results can be interpreted by the possibility that these proteins/SGG have backups for one another, as the fertilization process is of utmost importance for the maintenance of life in the next generation within a species [18]. Supporting this concept is the fact that sperm head surface proteins with ZP affinity exist together as high molecular weight complexes, which have direct ZP binding ability [19,20].

With sperm physiological events described above, it is logical to search for compounds that disable sperm fertilizing ability through the following processes: forward motility in non-capacitated sperm, and hyperactivated motility, acrosome reaction and sperm-ZP binding in capacitated sperm. However, targeting each individual event may be a complicated task, especially at the step of sperm-ZP binding in which numerous sperm surface molecules are engaged. Hypothetically, inhibiting sperm capacitation would result in inability of sperm to fertilize eggs. However, there are several challenges towards this approach. First, compounds used to inhibit sperm capacitation should be specific to only this sperm event, so as to minimize unrelated side effects. Second, sperm swim out from the seminal plasma in the vagina through the cervix and into the uterine cavity to undergo capacitation at different rates. Therefore, it is important that the tested compounds administered in the vagina can travel through the cervix into the upper part of the uterine cavity.

### 2.2. Unsuccessful Attempts to Develop the Spermicide, Nonoxynol-9, as a Microbicide

**Figure 1:**
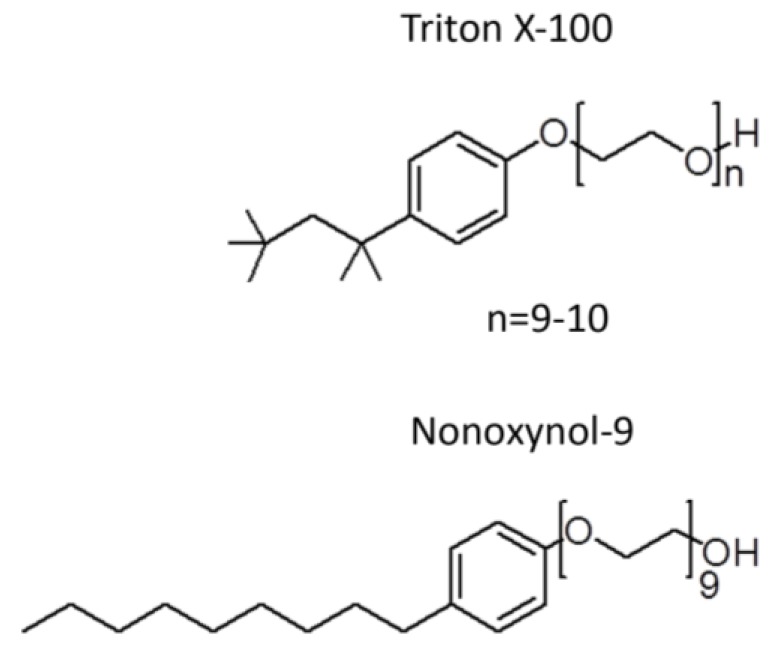
Similarity of the chemical structure of nonoxynol-9 and Triton X-100.

Since the search for spermicides started over 50 years ago before the significant unfolding of the molecular mechanisms on how sperm gain fertilizing ability, a simple Sander-Cramer assay based on sperm immotility was implemented at the time for spermicide screening. Compounds are considered as spermicides if they can completely inhibit motility of sperm in the diluted semen suspension within 20 s. However, these criteria are unlikely to represent the physiological events occurring during conception. First, motile sperm swim out from the liquefied semen in the vagina to enter the cervix and then the uterine cavity, while most of the seminal plasma is left in the vagina along with sperm that have much less progressive motility. Given this scenario, a spermicide needs to target those motile sperm with only residual amounts of the seminal plasma. In contrast, in the Sander-Cramer assay, semen is diluted three to four-fold with medium, leaving 20%–25% of seminal plasma in the sperm suspension. The seminal plasma contains high amounts of amyloid peptide complexes, which surround sperm in the ejaculate [21]. In general, this makes it hard for compounds added exogenously to reach the surface of sperm in the seminal plasma. As such, only compounds that have the strength to immediately disaggregate the amyloid complexes, such as harsh surfactants, can accomplish the task. Thus, the major compound identified by the Sander-Cramer assay for its spermicidal effects is nonoxynol-9 (N-9), which is a non-ionic detergent with a very similar structure to Triton X-100 (Figure 1). At 0.0075% to 0.012%, N-9 can irreversibly immobilize human sperm in a saline/medium diluted semen suspension (3-4 fold dilution) within 20 s [22,23,24], and therefore it has been used for more than 50 years as a spermicide in condom coating and in various vaginal contraceptive devices (*i.e.*, foams, suppositories, creams, gels and films). As a detergent, it is not surprising that N-9 exerts microbicidal effects *in vitro* against various types of STI-induced microbes as well as HIV [25,26,27,28,29]. N-9 formulated foam was further shown to prevent rhesus macaques from SIV and SHIV infection, albeit in a very small cohort of monkeys (<10) [30,31]. Nonetheless, these results paved the way to clinical trials on the anti-STI activity of N-9 in women. However, a systematic review using meta-analyses of 10 respectable clinical trials, including 5909 participating women, failed to demonstrate significant protective effects of N-9 against a wide range of STI-induced microbes including *Neisseria gonorrhoeae*, *Chlamydia trachomatis*, HIV, *Candida albicans* and *Trichomonas vaginalis* [32]. In fact, in one clinical trial, the HIV infection rate was shown to increase twofold in women using N-9 gel, compared with those without any application [33] and in another clinical trial, the gonorrhoea infection rate was shown to be higher in N-9 vaginal gel users [34]. These increased rates of STI are likely due to the “detergent” action of N-9 on cervicovaginal surface membranes. In most of the clinical trials, vaginal toxicity including irritation, ulcerations, histological inflammation as well as vulvitis was observed in women who were vaginally exposed to N-9 in various forms (e.g., N-9 formulated gels, films, sponges and suppositories, and N-9 coated condoms) [35,36,37,38,39,40]. Vaginal epithelial disruption was in fact observed by colposcopy in women frequently using N-9 vaginal suppositories [41]. Similar results were observed in mice intravaginally inoculated with N-9 [42]. These cervicovaginal epithelial disruptions likely facilitate the entry of HIV and possibly also other microbes through the genital mucosa. In addition, specific cytokines are produced in N-9 using women with cervicovaginal inflammation, resulting in recruitment of immune cells, which are typical HIV host cells, to the vaginal lumen. Essentially, this enhances HIV replication [43]. Currently, N-9 is not promoted for its use as a microbicide and most established pharmaceutical companies have stopped coating condoms with N-9. However, all vaginal products, such as foam and cream, which contain N-9 (can be up to 28%), are still available over the counter for contraceptive use, although the products contain a warning message against their use in women who are prone to HIV and other microbe exposure. Scientifically, it is hard to understand why N-9, a detergent, was even developed as a spermicide with a false hope that its disruption effect would be specific to sperm membranes, and not cervicovaginal epithelial cell membranes. Nonetheless, the adverse outcomes of the attempts to develop N-9 as vaginal microbicides provide a valuable lesson to the scientific/medical community. Namely, the integrity of the female reproductive tract must be considered for all future microbicide/spermicide development.

## 3. Antimicrobial Peptides as Spermicides

An alternative approach to search for compounds with both spermicidal and microbicidal actions can be taken by screening for spermicidal properties of known microbicidal agents. However, conventional antibiotics must be excluded in this pursuit due to the growing antibiotic resistance [44,45]. In this regard, antimicrobial peptides (AMPs) have been appropriately considered, as they are small peptides (<10 kDa) produced by all domains of life as part of innate immunity. In humans and eutherian mammals, AMPs are produced by neutrophils and other immune cells as well as epithelial cells of various tissues especially those that are connected or exposed to an external environment (e.g., genitourinary tract, lung, skin) [46,47]. As natural compounds, resistance against the microbicidal activities of AMPs is less anticipated and AMPs are considered to be the next generation of anti-infectives [45]. The broad spectrum activity against Gram positive and Gram negative bacteria, fungi, viruses and certain protozoa is another attractive property of AMPs [46,47,48]. In addition to the direct microbicidal properties, AMPs can abrogate the action of lipopolysaccharides (LPS), a pathogen associated molecular pattern (PAMP) from Gram negative bacteria [47], through their affinity for LPS [49,50,51]. Therefore, the binding of LPS to the host cell surface Toll-like receptor 4 and subsequent cell signalling events that lead to inflammatory responses cannot occur [52]. AMPs can also act as immune modulators with various positive consequences to the target cells (reviews [53,54,55]). For this reason, AMPs are also referred to as host defence peptides [46,55]. Immunomodulatory effects of AMPs, which are relevant to the health of the lower female genital tract tissues include wound repair, angiogenesis and cell proliferation [46,48,52,56,57,58,59], as these processes would be beneficial to the vaginal mucosa after intercourse that often induces abrasion to the mucosal surface.

Most of AMPs are cationic and amphipathic peptides. To date, over 2500 natural AMPs with diverse sequence, structure and property have been archived in the APD3 database (http://aps.unmc.edu/AP/ [60]). Based on the types of secondary structures, AMPs are categorized into four classes (α, β, αβ, and non-αβ) [61]. Because less than 12% of the AMPs in the APD3 database have a 3D structure, covalent bonding patterns of polypeptide chains are also used to categorize AMPs into four classes: linear (UCLL, with no covalent bonds between different amino acids in the polypeptide chain, e.g., human cathelicidin LL-37 and frog magainins), side-chain linked (UCSS, covalent bonds between different amino acid side chains, e.g., disulfide bonded defensins and lanthionine ether bonded lantibiotics), sidechain-backbone linked (UCSB, such as those present in bacterial lasso peptides), and backbone-backbone connected circular peptides (UCBB, such as those present in plant cyclotides) [62]. In addition, specific properties have been used to define AMP families. For example, cathelicidins are AMPs, where their precursors contain a conserved cathelin domain in the N-terminal region [46]. Examples include LL-37 in humans, CRAMP in mice, indolicidin in cows. Another family of AMPs containing disulfide bonds is called “defensins”, which are sub-classified to the α, β and θ groups. The α- and β-defensins are differentiated from each other based on the positions of the disulfide bonds (α having C^I^–C^VI^, C^II^–C^IV^ and C^III^–C^V^, whereas β having C^I^–C^V^, C^II^–C^IV^ and C^III^–C^VI^). So are θ-defensins, which possess three different disulfide bonds (C^I^–C^VI^, C^II^–C^V^ and C^III^–C^IV^). Interestingly, most of defensins with known 3-D structure (α: HNP-1, HNP-2, HNP-3, HNP-4, HD-5 and HD-6; β: hBD-3; θ: RTD-1) have β-sheet secondary structure, while hBD-1 (human β-defensin-1) contains both α-helix and β-sheet structures. In addition, “bacteriocins” is used to define AMPs produced by bacteria. Notably, a number of bacteriocins contain intramolecular lanthionine or methyl lanthionine ethers (formed through thioether linkages between serine/threonine and cysteine); such bacteriocins are referred to as lantibiotics and lantipeptides depending on whether they have antibacterial activity [63]. In addition, some bacteriocins such as gramicidin A contain d-amino acids [64]. Both lanthionines and d-amino acids contribute to the 3-D structure of bacteriocins [65]. It is notable that a number of bacteriocins differ substantially from cationic cathelicidins and defensins. For example, gramicidin A has a net charge of zero and consists of essentially all hydrophobic amino acids, allowing it to insert into bacterial membranes as an ion channel. In addition to structural categorization, APD3 classifies AMPs according to their functions (e.g., antimicrobial (as expected), anti-HIV, antimalarial, wound healing). Relevant to this review is the spermicidal activity of certain AMPs, at least *in vitro*. These AMPs are referred to as spermicidal AMPs.

Being positively charged, AMPs first bind to anionic molecular components of the cell wall of Gram positive bacteria (*i.e.*, lipoteichoic acid) and outer membrane of Gram negative bacteria (*i.e.*, LPS) [66]. This initial binding results in destabilization of these outer surfaces, thus allowing AMPs to reach and bind to anionic molecules of the bacterial cell membrane (*i.e.*, phosphatidylglycerol (PG) and cardiolipin (CL)). Initially, AMPs may lie horizontally through their affinity for PG and CL on the bacterial cell membrane. Since AMPs are amphiphathic, clusters of their hydrophobic amino acids would then interact with the hydrocarbon chains of lipids in the membrane bilayers. This may result in the vertical insertion of AMP peptides into the lipid bilayer. AMPs adjacent to each other also tend to polymerize through the interaction of their hydrophobic domains. Alternatively, these peptides may intersperse in the membranes by preferentially interacting with anionic lipids. All of these could lead to pore formation in the bacterial cell membrane and the eventual loss of cellular homeostasis, and finally death [46,48,67]. In addition, some AMPs can inhibit bacterial cell wall synthesis [67], while others can enter the bacteria to bind to their ribosomes, resulting in inhibition of protein translation [60]. The fungicidal, virucidal and protozoacidal mechanisms, however, are less understood [48].

**Figure 2:**
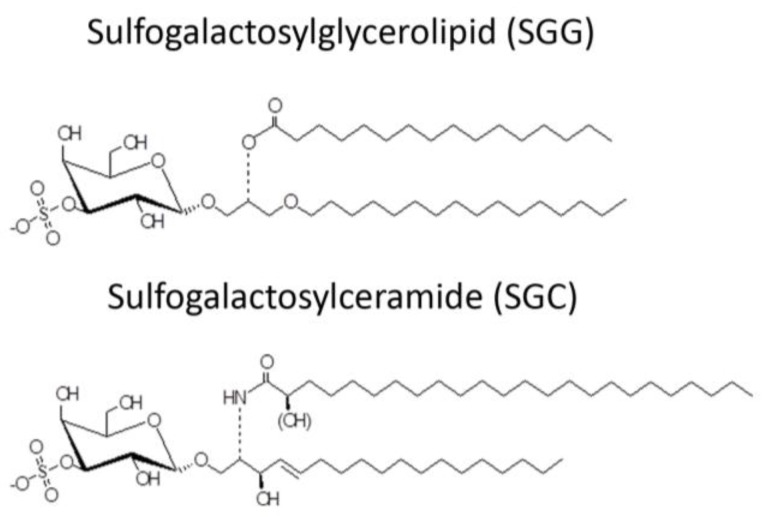
Chemical structure of sulfogalactosylglycerolipid (SGG) and sulfogalactosylceramide (SGC).

The desire to use AMPs as spermicides has been in place for decades, although in the early phase of work, the selective action of AMPs on sperm and not on epithelial cells of the vagina (the most logical site for their administration) may not have been well considered. Mammalian sperm are known to have a negatively charged surface [68,69] in the head region. This is due to the specific presence of SGG in the outer leaflet of the plasma membrane. The level of SGG is at 10 mole% of total sperm lipids with the main molecular species having C16:0 in both its *sn-1* alkyl chain and *sn-2* acyl chain [70,71] (Figure 2). SGG is a structural analog of sulfogalactosylceramide (SGC, aka sulfatide) (Figure 2), which is known to be an integral component of membrane lipid rafts. Likewise, SGG is a major lipid in sperm lipid rafts; it exists together with its lipid binding partner, cholesterol [72,73]. Our lab has shown that lipid rafts in capacitated sperm, housing a number of ZP binding proteins [19,20,74], are surface platforms on sperm for the ZP binding [72]. SGG itself also has a direct affinity for the ZP, contributing to the ZP binding ability of sperm lipid rafts [15,16]. It is expected that positively charged molecules, including AMPs, would interact electrostatically with SGG on the sperm head surface and thus reduce the sperm-ZP binding process. In addition to SGG, polysialyated glycoproteins found in the sperm plasma membrane endow negative charges to the sperm surface [75,76].

Table 2 lists all spermicidal AMPs described in the literature and the antimicrobial peptide database (http://aps.unmc.edu/AP/ [60]), with their sequences and relevant biochemical properties described in Table 3.

In addition to these 12 AMPs, cecropin D2A21 was mentioned but without any data on its spermicidal effects in the publication describing its chlamydiacidal property [111]. Seven of these AMPs are produced in humans and animals, *i.e.*, LL-37-humans; maximin 1, maximin 3, magainin 2, dermaseptin S1 and dermaseptin S4-lower vertebrate animals (frogs and toads); and sarcotoxin Pd-invertebrates (insects). The other five AMPs (nisin A, pediocin CP2, gramicidin A, subtilosin A and lacticin 3147) are bacteriocins produced by various bacteria species. Interestingly, gramicidin A has been used for decades in humans in Russia [105], although the scientific and medical details of this use are not available in the literature. Except for our work on LL-37, the screening for the spermicidal action of AMPs listed in Table 1 was based primarily on their ability to immobilize sperm in the suspension that still contained 20%–25% of seminal plasma within 20 s of treatment (Sander-Cramer test). As discussed above, this approach is likely an “overkill”, since motile sperm swim out of the seminal plasma and carry only residual amounts of seminal plasma into the cervix and uterine cavity. Rather “swim-up” sperm and “Percoll-gradient centrifuged”, prepared in a laboratory, would be more representative of motile sperm that swim through the cervix into the uterine cavity [23]. Swim-up sperm are simply prepared by overlaying semen placed in a tube with appropriate medium. Swim-up sperm are motile sperm that have swum up into the medium layer that still carry a small amount of seminal plasma [112]. Percoll-gradient centrifuged sperm are prepared by loading semen onto a discontinuous gradient of Percoll solutions (usually 45% and 90%) followed by centrifugation at ~600 g. Motile sperm have “clean” morphology (devoid of membranous vesicles and having compact chromatin) with a higher specific density than immotile/morphologically abnormal sperm, and therefore they sediment as a pellet, which can then be resuspended in a medium for experimental use. Percoll-gradient centrifuged sperm have the highest fertilizing ability [113] and therefore they should be used for the screening of a candidate spermicide. However, an assay for human sperm motility that is more physiologically representative is to observe the rate of sperm to swim through cervical mucus collected from women in the middle of their menstrual cycle (ovulation time) and placed in a capillary tube [7,114]. The cervical mucus fluid is viscoelastic due to the abundance of proteo-glycosaminoglycans. Sperm must have sufficient motility force as well as biochemical components to digest the proteo-glycosaminoglycan networks in order to swim through the cervical mucus column, a situation that does not exist in sperm resuspended in medium [11,115]. This sperm-cervical mucus assay was performed with gramicidin A treated human sperm. This assay, however, is impractical to be used for screening of spermicide compounds due to the restriction in obtaining sufficient quantities of mid-menstrual cycle cervical mucus from women.

Among all spermicidal AMPs listed in Table 2, LL-37, magainin 2 and nisin A are the only AMPs with confirmed *in vivo* contraceptive effects in experimental animals. The outcome of zero pregnancies is considered the gold standard indicator of contraceptive effects of these AMPs. Our group performed this *in vivo* work on LL-37 in mice [79], whereas Reddy & Aranha’s group examined the effects of nisin A in rats and rabbits [98,99], and magainin 2 in rabbits and monkeys [82,84]. Experimentation in animal models is essential in the screening of vaginal spermicides and/or microbicides, since it is a means not only to confirm the spermicidal/microbicidal effects *in vivo* but also to determine whether there are any adverse effects of the compounds on the female reproductive tract. Selection of appropriate animals for these *in vivo* studies is also critical for the next clinical trials in humans. Monkeys are animals with reproductive physiology closest to the human system. However, one may only use a restricted number of monkeys for each trial. In addition, it is important to preserve the female monkey lives after the experiment. Therefore, the examination of any adverse effects on the reproductive system in female monkeys can only be done in an indirect manner (e.g., assessing types and quality of cells from vaginal smears). Such is the case for the study on *in vivo* spermicidal effects of magainin 2 in monkeys [82]. In contrast, histology of the female reproductive tract can be evaluated directly in smaller animals including rabbits, rats and mice following their sacrifice and tissue removal for fixing and processing. Histology images should be explicitly shown such as those described in the studies for LL-37 in mice [79] and nisin A in rabbits [100]. However, interpretation of results from spermicidal and microbicidal studies in female rabbits, rats and mice must be made with caution that the reproductive systems in these animals differ from that of humans in a number of aspects. While insemination in rabbits occurs in the vagina like the situation in humans, rabbit females do not have a reproductive cycle (estrus or menstrual cycle) with peaked estrogen, which enriches the vaginal/cervical secretion and microflora [116]. Therefore, rabbit vaginal microflora are rather simple and in fact contain only residual amounts of lactobacilli [116], which are found abundantly in the human vagina and responsible for acidifying the vaginal luminal milieu due to lactate production [117]. The pH in the rabbit vagina is in the neutral range [118] in contrast to the pH 4 in the human vagina [119,120]. With no defined reproductive cycle, ovulation in rabbits is not hormonally regulated but rather induced by coitus [121], a process so markedly different from human ovulation. The reproductive processes in rats and mice, as well, differ from those in humans in a number of aspects despite the fact that rodents do have a reproductive estrus cycle. Although rodent semen is first deposited in the vagina, sperm together with seminal plasma are swept into the uterus within minutes [11]. In addition, the mouse vaginal pH is at ~6.5, more basic than that of the human vagina. This is due to lower numbers of lactobacilli colonies in the rodent vagina [122]. Regardless, rodents are less expensive to purchase and maintain and the *in vitro* fertilization procedures in rodents are well described, allowing the assessment of candidate spermicides for their direct inhibitory effects on fertilization *in vitro*. While it is logical that various animal models should be used to validate spermicidal and microbicidal effects of the candidate compounds, human vaginal and cervical cell lines as well as reconstructed human vagina models should also be employed in the spermicide and microbicide *ex vivo* studies. The former include immortalized cell lines derived from human vaginal epithelia (Vk2/E6E7), human ectocervical epithelia (Ect1/E6E7) and human endocervical epithelia (End1/E6E7), all established by Deborah Anderson, Brighams and Women’s Hospital, Inc. [123], and available from ATCC. For the latter, MatTek Inc. has produced various types of organotypic vaginal-ectocervical tissue models through reconstruction of human vagina and cervix tissues [124]. Vk2/E6E7, Ect1/E6E7 and End1/E6E7 cell lines have been widely used for the studies of candidate microbicides [125,126,127]. Likewise, MatTek human vaginal-ectocervical tissue models have recently been used for studies on the properties of potential vaginal anti-HIV agents [128,129,130]. To date, there are no publications on the use of immortalized Vk2/E6E7, Ect1/E6E7 and End1/E6E7 cells for studies of spermicidal AMPs. MatTek organotypic vaginal-ectocervical tissue models were used only in subtilosin A study (Table 2). On the other hand, HeLa cell lines (derived from cervical cancer cells obtained from Henrietta Black over 60 years ago [131]) were used for evaluating cytotoxicity of dermaseptin S4, sarcotoxin Pd and nisin A (see Table 2). However, HeLa cells are highly transformed and may not have the expected properties of human cervical epithelial cells [131].

As discussed above, the acidic pH in the healthy human vagina is attributed to the presence of commensal lactobacilli. The vaginal acidity is essential in inhibition of unwanted proliferation as well as infection of pathogenic microbes such as *Gardnerella vaginalis*, *Prevotella bivia* and HIV [132]. Depletion of lactobacilli in the vagina leads to bacterial vaginosis. In this regard, caution must be taken in the selection of AMPs with no microbicidal effects on lactobacilli for further development into vaginal microbicides. This is the case for nisin A, which has been used as a food preservative for human consumption and shown to be an effective contraceptive in rabbits and monkeys ([98,99], Table 2). However, nisin A is not a good candidate to be developed into a vaginal microbicide, since it exerts microbicidal activity on vaginal *Lactobacillus spp* [132,133].

While the microbicidal effects of AMPs in general are of a broad spectrum, it is most desirable to search for spermicidal AMPs that also have anti-HIV properties. Among spermicidal AMPs listed in Table 2, LL-37 [134,135,136], maximin 1 [80], maximin 3 [80], dermaseptin S1, dermaseptin S4 [137] and gramicidin A [105] have been shown to possess anti-HIV properties. Therefore, they should be considered for further research studies and development into vaginal contraceptives/microbicides. Most of these HIV infection studies were performed using HIV lab strains in CD4^+^ T cells, T-cell leukemia cell lines or T4 lymphoblastoid cell lines, and in some cases monocyte derived dendritic cells. In order to validate the physiological significance of the anti-HIV properties of these AMPs, work should be comprehensively repeated using dual tropic clinical isolates of HIV at sufficiently high titers. In addition, the inhibitory effects of the candidate AMPs on HIV infection in the human cervicovaginal epithelium should be directly assessed using MatTek human vaginal-ectocervical tissue models. On the other hand, since gramicidin A has been used as a spermicide in Russia [105], a population study should be carried out to determine whether women who have been using gramicidin A vaginally and are more prone to exposure to HIV have a lower rate of HIV infection.

The 3D structures of a number of spermicidal AMPs or their close homologs have been determined by NMR spectroscopy. These include human LL-37 [77], frog maximin 4 (homolog of maximin 1) [81], magainin 2 [83], dermaseptin S4 [91], bacterial nisin A [138], gramicidin A [64], subtilosin A [108] and lacticin 3147 (chain A1 and A2) [110].

**Figure 3:**
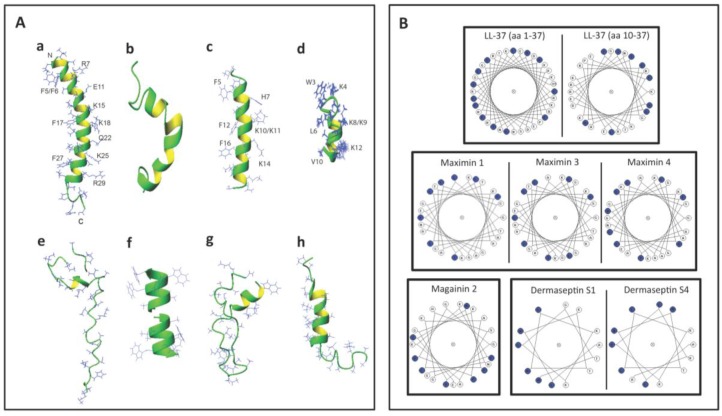
Structures of selected antimicrobial peptides with spermicidal activity annotated in the APD3 [142]. (**A**) 3D structures shown as ribbon diagrams of (**a**) human LL-37 (PDB ID: 2K6O); (**b**) frog maximin 4 (PDB ID: 2MHW); (**c**) magainin 2 (PDB ID: 1MAG); (**d**) a close analog of truncated dermaseptin S4 (amino acids 1-13; PDB ID: 2DD6); (**e**) bacterial nisin A complexed with lipid II (PDB ID: 1WCO); (**f**) gramicidin A (PDB ID: 1MAG); (**g**) subtilosin A (PDB ID: 1PXQ); and (**h**) lacticin 3147 (structural co-ordinates were provided by Dr. John Vedaras, University of Alberta, according to his published work [110]). Except for gramicidin A, the N-terminus of the peptide is positioned at the top. In the case of gramicidin A (**f**), its dimer is shown. The two N-termini of each dimer are positioned next to each other in the middle, whereas the C-termini are exposed and their four tryptophans approximate the lipid head group regions of the lipid bilayers for membrane positioning and ion channel conductance (**f**). Note that the C and N-termini of subtilosin A approximate in the structure (**g**). The side chains of human LL-37, magainin 2, and the dermaseptin S4 analog are selectively labeled to illustrate the amphipathic nature of these AMPs. Images were generated using MOLMOL [143]. (**B**) Helical wheel projections of selected spermicidal AMPs expressed in eukaryotes: LL-37, maximin 1, maximin 3, maximin 4, magainin 2, truncated dermaseptin S1 and dermaseptin S4. All of these AMPs show an amphipathic structure, with hydrophobic amino acids (blue circles) organized in approximately one half of the wheel and the hydrophilic residues in the other half. For LL-37, the wheel projections are shown for both the whole LL-37 sequence (amino acids 1–37) and the sequence from amino acids 10–37. This is because the helical structure of the whole LL-37 sequence has a kink at Ser^9^. The LL-37 peptide (amino acids 10-37) actually shows a better distribution of hydrophobic amino acids in one half of the helical wheel. Although maximin 4 has not been shown for the direct microbicidal effects, as demonstrated for maximin 1 and maximin 3, its helical wheel projection is shown herein to corroborate its 3D structure shown in (**A**) and also for a comparison with the wheel projections of maximin 1 and maximin 3. The wheel projections of truncated dermaseptin S1 and dermaseptin S4 are both for their truncated sequence (amino acids 1-13). Again, this is to corroborate the 3D structure of the close analog of the truncated dermaseptin S4 peptide shown in (**A**).

Figure 3A shows the 3D structures of these AMPs. Interestingly, LL-37, maximin 4, magainin 2, dermaseptin S4 and lacticin 3147 chain A2 contain an alpha helical structure in a significant length of their sequences. A short helix also exists in subtilosin A and nisin A (Figure 3A). However, lacticin 3147 chain A1 (which has a minimal spermicidal activity) does not have an alpha helical structure [110] and is therefore not displayed in Figure 3A. On the other hand, gramicidin A has a β-helical structure (Figure 3A). The helical wheel projections of LL-37 (both the whole peptide and the sequence from amino acids 10-37), maximin 1, maximin 3, maximin 4, magainin 2, dermaseptin S1 and dermaseptin S4 (all from humans and animals) reveal an amphipathic feature of the helix, with hydrophobic amino acids enriched in approximately one half of the top view helical circle and hydrophilic amino acids in the other half (Figure 3B). Such an amphipathic feature can also be seen in Figure 3A, especially for LL-37, magainin 2, and dermaseptin S4 peptide analog. It is remarkable that both LL-37 and magainin 2 possess 3-4 aromatic phenylalanines on the hydrophobic surfaces. NMR studies have demonstrated that the four phenylalanines all interact with acyl chains of anionic PGs [77]. This amphipathic helical structure of AMPs may be required for spermicidal activity. If this is proven, this requirement would be analogous to that needed for AMPs for full exertion of microbicidal activity [135,139,140]. 

Bacteriocins utilize specific features to maintain their 3D structure. Lacticin chain A1 does not have any helical structure but its 3D confined structure is attributed to the existence of both d-amino acids and lanthionine ethers in its sequence [110]. These two structural components also exist in lacticin 3147 chain A2 with an apparent alpha helical structure [110]. Nisin A and subtilosin A also contain lanthionine ethers [46,141], which contribute to their 3D structure (Table 3). On the other hand, gramicidin A does not contain any lanthionines but it possesses five D-amino acids in its 15-mer sequence (Table 3). This unique feature endows gramicidin A a β-helical structure, which is further stabilized by dimerization, and this allows gramicidin A dimer to form a cation channel in lipid bilayers [64]. The channel and pore formation in the membranes by gramicidin A and other spermicidal bacteriocins like subtilosin A and lacticin 3147 chain A1 is unlikely to be initiated with the electrostatic binding between these AMPs and the negatively charged surface molecules of the microbes, since these AMPs have a net charge of zero or even minus values (Table 3). A close look at the helical regions in these bacteriocins (Figure 3E, G, and H) reveals a lack of amphipathic nature observed with human LL-37, magainin 2 and dermaseptin S4 (Figure 3A,B).

## 4. LL-37, the Most Promising Spermicidal AMP

Cationic antimicrobial peptide LL-37 is coded by the only human cathelicidin gene [46,47,144]. LL-37 or its very close homologs are also present in non-human primates. Chimpanzees possess LL-37 identical to the human sequence, whereas in Gorillas and orangutans, two and three spots in the human LL-37 sequence are replaced by other amino acids [145]. The name “LL-37” denotes a peptide that contains 37 amino acids with the Leu-Leu sequence at the N-terminus. LL-37 is produced as a propeptide, hCAP-18, by neutrophils [50,146], other immune cells [147,148], normal and inflammatory skin cells [149,150,151], and epithelial cells of various tissues, especially those that connect with the external (male reproductive tract [152,153], urinary tract [154,155], gastrointestinal tract [156], lung [157,158], gingiva [159], eye [160], nasal cavity [161,162]) (for reviews see [149,163,164]). Of significance to this review is the expression of hCAP-18 by the human epididymal epithelial cells followed by its secretion into the epididymal lumen. This makes hCAP-18 a component of seminal plasma with a physiological concentration range of 2 to 10 μM [152,153,165]. hCAP-18 (MW:16442) is expressed by neutrophils and released during degranulation together with proteinase 3, which immediately processes hCAP-18 at physiological pH into LL-37 (MW:4493) with full microbicidal activity [166]. In skin, LL-37 is generated from hCAP-18 through the proteolytic activity of kallikrein 5 also at the neutral pH. Both kallikrein 5 and kallikrein 7 (also present in the skin surface) then further cleave LL-37 into smaller fragments (*i.e.*, RK-31, KS-30, KS-22, KR-20, LL-29), which have higher antimicrobial activity than LL-37 [151]. However, the processing of hCAP-18 is more complicated. It does not occur in the male reproductive tract or in the ejaculate. Sorensen *et al.* [153] identified gastricsin, secreted from the prostate gland and thus also a component of seminal plasma, to be the enzyme responsible for processing hCAP-18 in seminal plasma into ALL-38 (LL-37 + Ala at the N-terminus) at a pH optimum of approximately 4. Therefore, gastricsin cannot function in seminal plasma, which has a high buffering capacity at neutral pH. Following ejaculation in the vagina, seminal plasma changes the normally acidic pH of the vagina (pH 4) into the neutral range. It takes 2-6 h post-ejaculation for the vaginal lumen to resume its acidic pH and it is only at this time that gastricsin becomes active to process hCAP-18 into ALL-38, with microbicidal activity [153].

**Figure 4:**
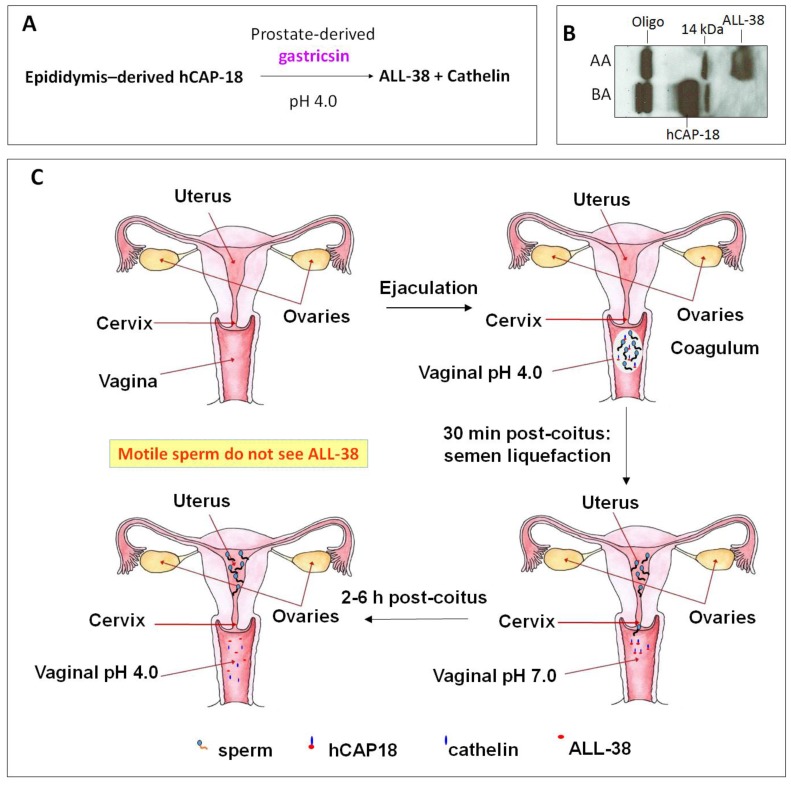
ALL-38 is processed from hCAP-18 post-ejaculation. (**A**) Processing of hCAP-18 to ALL-38 by gastricsin occurs at the optimum pH of 4. Although both hCAP-18 and gastricsin are seminal plasma components, this processing does not occur in the seminal plasma due to its high buffering capacity at neutral pH. (**B**) Immunoblotting showing that processing of hCAP-18 to ALL-38 can occur in acidified seminal plasma. Anti-LL-37 antibody used in immunoblotting was produced against the whole LL-37 sequence [79] and therefore, it recognized only ALL-38 and hCAP-18, but not cathelin. Before acid treatment (BA), seminal plasma contains hCAP-18 and its oligomers (Oligo; ~60 kDa), as well as a 14 kDa band (presumably a cleaved product of hCAP-18). Upon acidification of seminal plasma with HCl to pH 4, hCAP-18 is processed to ALL-38; this is due to the activation of gastricsin. AA = after acid extraction. (**C**) ALL-38 is produced 2–6 h post-ejaculation. Upon ejaculation, seminal plasma neutralizes the vaginal pH, and it takes 2–6 h post-ejaculation for the vagina lumen to resume its acidity. Only at this time, ALL-38 is produced from hCAP-18 via gastricsin activity. However, immediately after semen liquefaction (30 min post-coitus), motile sperm in the ejaculate swim into the uterine cavity. Therefore, motile sperm are never exposed to ALL-38. The drawing is based on Sørensen *et al.* [153].

However, immediately after semen liquefaction, typically 30 min post-ejaculation, sperm instantaneously swim out from seminal plasma through the cervix into the uterine cavity. Only feeble sperm are left behind together with seminal plasma in the vagina. Therefore, motile sperm with fertilizing ability are never exposed to ALL-38, which is generated long after their movement from the vagina (Figure 4). It is likely that the production of ALL-38 is meant for protection of the vaginal epithelium against microbes introduced during intercourse [167]. As a very close analog of LL-37, ALL-38 should possess all properties described for LL-37. For example, they have identical antibacterial activity against all the organisms tested [153]. LL-37 has been shown for its direct microbicidal effects against numerous Gram negative and Gram positive bacteria, yeast and viruses (including HIV) (Table 4), but not on lactobacilli [168,169,170], which are essential for maintaining acidic pH and thus health of the vagina (see Section 3).

Besides the direct microbicidal activity, LL-37 possesses other properties including anti-endotoxin activity [49,50,51], immunomodulation (reviews [53,54,55]), angiogenesis [59] and wound healing [56,57,58]. If the anti-endotoxin and immunomodulatory properties are confirmed in the female reproductive tract system, it will strengthen the possibility that LL-37/ALL-38 can be used as vaginal microbicides that can clear infection and minimize infection-associated inflammation. Angiogenesis and wound healing properties would also aid in the repair of minor vaginal tissue damages occurring during intercourse. Further studies on LL-37’s direct microbicidal effects must also be carried out in all microbes that are causes of STIs and vaginitis, as well as urinary tract infection (UTI). The vagina and its normal microbiota represent an important barrier against uropathogenic bacteria (Table 1). Perturbations of the normal vaginal microbiota, such as depletion of lactobacilli, can promote colonization of uropathogens, such as uropathogenic *E. coli* (UPEC), within the vagina [205]. The vagina can then become an extra-urinary uropathogen reservoir and in turn increase the risk of UTI [206].LL-37 exerts microbicidal effects on most of the STI-inducing microorganisms including HIV (causing life threatening AIDS with no cure), HSV-1 (causing genital herpes with no cure), *Neisseria gonorrhoeae* (causing gonorrhoea, curable), *Treponema pallidum* (causing syphilis, curable) and *Chlamydia trachomatis* (causing cervicitis, salpingitis and endometriosis, curable) (Table 4). Although gonorrhoea, syphilis and chlamydia infection are curable, a number of complications are associated with these three STIs. They all increase the risk of infertility. A higher susceptibility to HIV transmission is also associated with gonorrhoea and syphilis. Salpingitis and oviductal tubal blockade caused by chlamydia infection can also lead to ovarian cancer. For gonorrhoea, resistance to antibiotics used for the treatment has increasingly become a problem. As listed in Table 1, LL-37 needs to be tested for its microbicidal activity against a number of additional microorganisms that cause STI (viruses:HPV, hepatitis A and C; protozoon, *Trichomonas vaginalis*) and bacterial vaginosis (*Gardnerella vaginalis*, *Bacteroides* spp., *Mycoplasma hominis* and *Mobiluncus* spp.), prior to its development as a vaginal microbicide. Further testing against uropathogenic microorganisms (Table 1) will also allow LL-37 to be developed for therapeutic and prophylactic uses for urinary tract infection in this system. LL-37 formulated gel administered into the vagina would exert microbicidal action on uropathogenic microbes that opportunistically form a reservoir in the vagina. The vaginal secretion, which can travel upwards into the urinary tract, would also likely contain LL-37 released from the gel, which then can fight against microbes in this tract (see the list in Table 4).

The majority of sperm co-existing with seminal plasma, which contains 2-10 μM of hCAP-18 [153], remain motile in ejaculated semen of fertile donors. Interestingly, despite the negatively charged surface of sperm and the overall positive charge of hCAP-18 (+6, pI = 9.25), hCAP-18 is present at a residual amount on human sperm [79]. However, since motile sperm with fertilizing ability are never exposed to ALL-38 produced from seminal plasma (Figure 4), it raises a possibility that ALL-38/LL-37 may have a deleterious effect on sperm, possibly after the deposition of ALL-38/LL-37 onto the sperm surface. Although hCAP-18/LL-37 is also produced by cervicovaginal epithelial cells, its amount is 1000x less than ALL-38 originated from seminal plasma (i.e., 1.3 nM [136] *versus* 2–10 μM [153], respectively). Therefore, we asked the question of whether LL-37 when added exogenously to a sperm suspension could bind to sperm with a deleterious consequence on their fertilizing ability. Also, if sperm-LL-37 binding did occur, was it dependent on the interaction of LL-37 with a negatively–charged SGG existing specifically on the mammalian sperm head surface? The latter question is relevant in terms of possible development of LL-37 into a spermicide with specificity to sperm and not to other somatic cells such as cervicovaginal epithelial cells. Our results revealed that LL-37 bound to SGG and its anionic lipid analog, sulfogalactosylceramide (SGC), as well as phosphatidylserine (also negatively charged), all immobilized separately in a well of a microtiter plate, in a specific manner. *K*_d_ values of the binding of LL-37 to these three anionic lipids were 456, 157 and 24 nM, respectively. In contrast, LL-37 did not bind to neutral lipids, phosphatidylcholine and galactosylglycerolipid (GG—the desulfated form of SGG) [79]. Direct binding of LL-37 to Percoll-gradient centrifuged mouse and human sperm resuspended in medium was further demonstrated. For the reason described in Section 3, Percoll-gradient centrifuged sperm were first used in all studies, and in the case of human sperm replicate experiments were performed with swim-up sperm. The partial dependence of LL-37-sperm interaction on SGG on the sperm surface was then demonstrated by a decrease in LL-37 binding to sperm that were pre-incubated with anti-SGG antibody [79]. Pretreatment of capacitated mouse sperm with 3.6 μM LL-37 resulted in a complete loss of sperm ability to fertilize eggs *in vitro*. This 3.6 μM of LL-37 is equivalent to the concentration of SGG in the mouse sperm suspension used for the treatment [71,79], further corroborating the concept that SGG was involved in LL-37 binding. Notably, 100% of mouse sperm treated with 3.6 μM LL-37 became immotile within 5 min of treatment, and this result would be one of explanations for the inability of these LL-37 treated sperm to fertilize eggs *in vitro*. Likewise, human sperm lost their motility when treated with LL-37, although 10.8 μM of LL-37 was needed for the majority of sperm to become immotile. Furthermore, when non-capacitated sperm and partially capacitated sperm, both human and mouse, were treated with the same LL-37 concentrations as used for fully capacitated sperm, the inhibition of their motility was similarly observed [79]. Non-capacitated sperm, partially capacitated sperm and fully capacitated sperm were prepared in the lab by resuspending sperm in the medium without bicarbonate, calcium and albumin, with bicarbonate and calcium but no albumin, and with all of these three components, respectively. The prepared non-capacitated sperm represent sperm that have just swum out from the seminal plasma *in vivo*, whereas partially capacitated sperm resemble those that are swimming through the cervix. Finally, the prepared capacitated sperm are equivalent to sperm that have swum into the uterine cavity and their cholesterol is induced to release by albumin and HDL present in the uterus [8,207]. 

The loss of sperm motility upon LL-37 treatment can be from the direct interaction of LL-37 with the axoneme, the motility apparatus, in the sperm tail [8]. However, this possibility is discounted by the lack of binding of LL-37, added exogenously to the sperm suspension, to the sperm tail [79]. It is also unlikely that the mechanism of the effects of LL-37 on sperm motility is through its direct effect on CatSper, the cation channel responsible for sperm hyperactivated motility [12], since LL-37 also induces immotility in non-capacitated sperm, which normally do not have hyperactivated motility patterns. Rather, immotility of LL-37 treated sperm may come from the loss of intracellular homeostasis due to the LL-37 induced damage on the sperm surface, a situation that is parallel to that observed on the microbial membrane [46,67]. This postulation was confirmed by the observation that LL-37 treated sperm became positively stained with Sytox Green (a membrane impermeable DNA fluorescent dye), an indication that the surface membranes of these treated sperm were compromised. Pharmacokinetic studies further indicated that the sperm plasma membrane damage induced by LL-37 occurred prior to the loss of sperm motility. Finally, LL-37 treated sperm became acrosome-reacted, thus markedly lowering their ability to bind to the egg. Transmission electron microscopy confirmed that the damages on the sperm plasma membrane and the outer acrosomal membrane as well as the loss of the acrosome and in some cases the inner acrosomal membrane were the adverse effects of LL-37 on sperm [79]. In contrast, our unpublished results reveal that LL-37 at the spermicidal concentration range did not have any adverse effects to mouse eggs and embryos. Mouse eggs treated with 3.6 μM LL-37 were normally fertilized by sperm collected from a fertile animal, and mouse two-cell embryos treated with the same LL-37 concentration developed into blastocysts at the same rate as untreated two-cell embryos.

**Figure 5:**
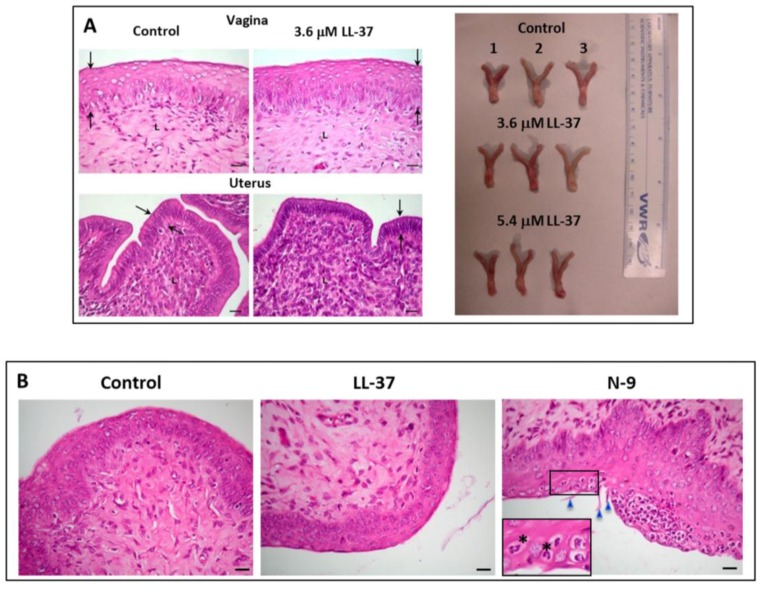
(**A**) Left panel: Normal histology of the vagina and uterus of female mice transcervically injected with LL-37 + sperm. The female reproductive tract tissues were collected for fixing and paraffin embedding one day after the transcervical injection of LL-37 + sperm, or sperm alone (control). Sections of the vagina and uterus revealed that the vaginal stratified epithelial cell layers and the uterine single epithelial cell layer (denoted as the area between the two arrows) as well as the corresponding lamina propria of the LL-37 treated and control animals did not differ from each other. Bar = 20 μm. This image was taken from our published article [79]. Right panel: The anatomy (shape, dimension and color) of the vagina/cervix and uterus, dissected from females injected with LL-37 (3.7or 5.4 μM) + sperm (n = 3 each) or sperm alone (control) (n = 3) one day after the injection, was the same among the three groups of the animals. (**B**) Absence of immune cell recruitment to the vaginal epithelium of mice transcervically injected with LL-37 + sperm. Sections of the vagina were prepared as in (**A**). The vaginal epithelium and lamina propria of both the control and LL-37 treated mice similarly show minimal numbers of immune cells, indicating no recruitment of these cells to the vagina as a consequence of LL-37 injection. In contrast, when females were transcervically injected with 2% nonoxynol-9 (N-9), numerous neutrophils were recruited into the vaginal epithelium. The polymorphonuclear structure of neutrophils is apparent in the close-up image (denoted by asterisks). Triangles point to the N-9 induced rupture of the vaginal epithelial surface. Bar = 20 μm.

We have further demonstrated the loss of sperm fertilizing ability *in vivo*. Mouse sperm treated with LL-37 were transcervically injected into female mice naturally cycling to the estrous phase in the reproductive cycle. None of these female mice (*n* = 26) became pregnant, whereas pregnancy was observed in 92% of female mice (*n* = 26) injected with untreated sperm. Significantly, the female reproductive system of mice injected with LL-37 + sperm did not show any apparent changes, as compared with that of control females (injected with medium + sperm) (Figure 5A). In females that were exposed to LL-37, the histology of their vagina and uterus as well as the dimension, shape and color of their reproductive tissues did not differ from those of control females (unexposed to LL-37) (Figure 5A). There were no signs of immune cell recruitment (implicating inflammation) to the vaginal/uterine tissues of these LL-37 exposed females (Figure 5B). This was in contrast to female mice transcervically injected with 2% N-9, whereas numerous neutrophils were apparent in the vaginal epithelium (Figure 5B). In fact, female mice transcervically injected with sperm + LL-37 for three estrus cycles with no pregnancy outcomes could resume their fecundity as shown by pups delivered by these females following their natural mating with fertile males two weeks after the last transcervical injection with LL-37. 

**Figure 6:**
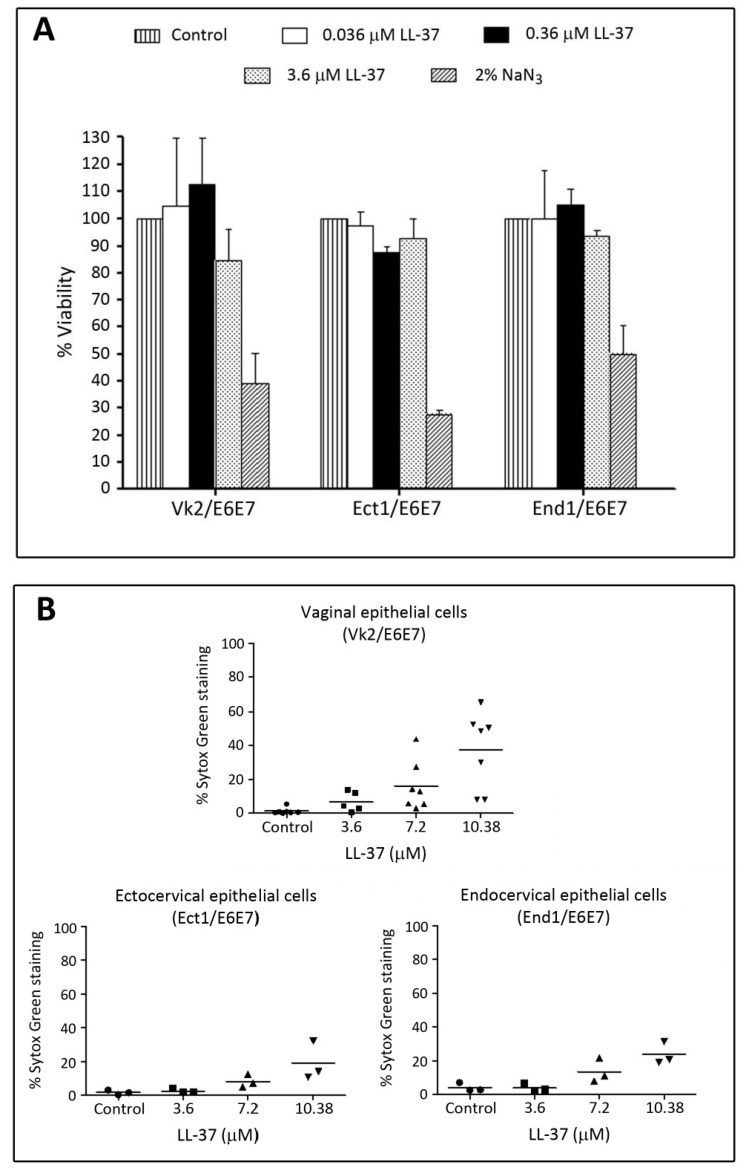
Low adverse effects of LL-37 on human vaginal and cervical cell lines. Vk2/E6E7 (vaginal), Ect1/E6E7 (ectocervical) and End1/E6E7 (endocervical) cells cultured as previously described [123] were treated with various concentrations of LL-37 (24 h, 37 °C, 5% CO_2_) and subjected to a cytotoxicity MTT assay (**A**) or a membrane intactness assay using Sytox (**B**). (**A**) Treatment of all three cell lines with LL-37 up to 3.7 μM did not change percentages of viable cells. In contrast, the percentages of viable cells were only 50% or less upon treatment with 2% NaN_3_. (**B**) Sytox Green staining was observed in less than 20% of all three cell lines upon treatment with LL-37 up to 3.7 μM. When the LL-37 concentration was increased to 10.8 μM, the numbers of Sytox Green stained cells became higher, although for both End1/E6E7 and Ect1/E6E7 cells, the percentages were still <20%. For Vk2/E6E7 cells, this percentage was 40%.

We have further confirmed that LL-37 had minimal adverse effects on the immortalized human cervicovaginal epithelial cell lines, *i.e.*, Vk2/E6E7 (vaginal), Ect1/E6E7 (ectocervical) and End1/E6E7 (endocervical) cells. Cytotoxicity MTT (3-(4,5-dimethylthiazol-2-yl)-2,5-diphenyltetrazolium bromide) assays showed no significant differences of percent viable cells of these three cell lines upon treatment with LL-37 at concentrations up to 3.6 μM (Figure 6A). Sytox Green incorporation assays for the membrane intactness of these cell lines revealed similar results. In all of these three cell lines, less than 10% of cells incorporated Sytox Green into their nuclei upon treatment with 3.6 μM. However, these percentages increased to 20, 25 and 40% in Ect1/E6E7, End1/E6E7 and Vk2/E6E7 cells, respectively, following treatment with 10.8 μM of LL-37 (Figure 6B). 

While the higher susceptibility to LL-37 in the vaginal epithelial cell line should be a matter of concern, the situation *in vivo* may not be as unfavourable as that observed *in vitro*. Cells of the upper layer of the vaginal epithelium continue to slough off into the lumen and it is possible that those cells with compromised membrane intactness due to LL-37 exposure may undergo this sloughing at a higher rate than normal cells.

In summary, we have shown that LL-37 possesses spermicidal effects in both human and mouse sperm with minimal adverse effects on the female reproductive tract epithelia. This spermicidal property with molecular mechanisms described above appears to be unique to LL-37. A number of defensin AMPs are secreted by epididymal epithelial cells (examples-human: HE2β1 (aka SPAG11D) [208], DEFB126 [209,210,211], DEFB118 [212,213], DEFB114 [214], DEFB1 [215]; -rodents: Bin1b (aka SPAG11E) [216,217], β -defensin 22 [218]) and some of them deposit onto the surface of transit sperm to endow fertilizing ability (e.g., HE2 β 1, DEFB126, DEFB118 and HBD1 in humans, and Bin1b in rodents). We have shown that both HE2 β 1 and Bin1b could bind to SGG/SGC *in vitro*, but sperm incubated with excess amounts (18 μM) of these two defensins were still motile with intact acrosome and in the case of mouse sperm that were treated with these β-defensins, they could still fertilize eggs at 50% control values (our unpublished results). 

The physiological significance of LL-37 as a microbicide and an immunomodulatory and wound healing peptide is well documented. In particular, LL-37 exerts microbicidal effects on a number of microbes responsible for STI, vaginitis and UTI (Table 4). With these microbicidal and spermicidal properties, LL-37 warrants further attempts to be developed as a vaginal contraceptive/microbicide. As a natural peptide, its repeated and long-term use will likely create minimal side effects and microbicidal resistance. Further studies, however, are needed in a number of aspects in potential application of LL-37 in the vagina. First, an effective method to deliver LL-37 into the vaginal lumen has to be established. Careful studies on any adverse effects of LL-37, following its repeated/long-term administration, must also be performed at both topical and systemic levels. Regardless, the cost of chemical synthesis of LL-37 for this use is a challenge, and the shortest truncated LL-37 peptide or its mimetic, which still bears spermicidal and microbicidal activity, has to be discovered. In a separate avenue, ultrashort cationic lipopeptides with high antibiotic activity [219,220,221] may be screened for spermicidal effects. However, the selectivity of the deleterious effects of these lipopeptides to sperm and not to the female reproductive tract epithelial cells must be considered. Nonetheless, success in finding the most cost-effective form of LL-37 as a vaginal contraceptive/microbicide will undoubtedly provide women with empowerment in having a healthy and safe sex practice—the ability to protect themselves from unwanted pregnancies and microbial infections.

## Figures and Tables

**Table 1:** Common microorganisms that cause sexually transmitted infection and genitourinary tract infection.

Sexually Transmitted Infection	Vaginitis	Urinary Tract Infection
**Viral Infection** Human immunodeficiency virus (HIV) Herpes simplex virus 1 & 2 (HSV-1 & HSV-2) Human papillomavirus (HPV) Hepatitis B and C **Protozoal Infection** *Trichomonas vaginalis* **Bacterial infection** *Chlamydia trachomatis* *Neisseria gonorrhoeae* *Treponema pallidum*	**Yeast Infection** *Candida albicans* **Protozoal Infection** *Trichomonas vaginalis* **Bacterial Vaginosis** *Gardnerella vaginalis* *Prevotella* spp. *Porphyromonas* spp. *Bacteroides* spp. *Peptostreptococcus* spp. *Mycoplasma hominis* *Ureaplasma urealyticum* *Mobiluncus* spp.	**Yeast Infection** *Candida albicans* **Bacterial infection** *Escherichia coli* *Klebsiella pneumoniae* *Enterococcus* spp. *Streptococcus agalactiae* *Proteus mirabilis* *Staphylococcus saprophyticus* *Viridans streptococci* *Klebsiella oxytoca* *Staphylococcus aureus*

**Table 2:** Spermicidal antimicrobial peptides: microbicidal, structural and spermicidal properties.

Peptide Name APD ^1^ ID UniProt ID PDB ID	Source	Microbicidal/Biological/Biochemical Properties	References for Spermicidal Effects	Remarks
LL-37 APD: AP00310 UniProt: P49913 PDB: 2K6O	Neutrophils, monocytes, lymphocytes, keratinocytes, epithelial cells of the lung, nasal cavity, genitourinary tract, gastrointestinal tract, ocular surface, and gingiva of *Homo sapiens*, Expression of LL-37 in *Pan troglodytes* (chimpanzee), *Macaca mulatta* (rhesus macaque) have also been described.	LL-37 exerts microbicidal effects on Gram positive and Gram negative bacteria, yeasts, *Candida albicans*, and HIV and other viruses, including those that cause STIs (see Table 4). In an aqueous solution, the structure of LL-37 is disordered. However, when it interacts with lipid membranes such as SDS and dodecylphosphocholine micelles, LL-37 adopts an alpha helical amphipathic structure, as revealed by NMR analysis with one side of the helix enriched in hydrophilic amino acids and the other side hydrophobic residues [77,78].	[79]	LL-37 completely inhibits human and mouse sperm motility within 5 min at 10.8 μM and 3.7 μM, respectively. This inhibition is likely due to the specific disruptive effects of LL-37 on sperm surface membranes, as shown by electron microscopy and Sytox Green (a membrane impermeable DNA dye) staining. In addition, LL-37 treated sperm become prematurely acrosome reacted, thus hindering them to effectively bind to the egg. These specific adverse effects of LL-37 on sperm are likely due to its affinity for the negatively charged sulfogalactosylglycerolipid (SGG) present selectively on the sperm surface. The contraceptive effect of LL-37 is further demonstrated in female mice. Females, naturally cycling to the estrous phase and transcervically injected with sperm + LL-37, fail to become pregnant, whereas pregnancy occurs in 92% of females injected with sperm alone. The reproductive tract tissues of the females administered with LL-37 appears to be normal like that observed in females unexposed to LL-37 [79].
Maximin 1 APD:AP00058 UniProt: P83080 PDB:None and Maximin 3 APD:AP00060 UniProt: P83082 PDB:None	Skin: Chinese red belly toad *Bombina maxima*	Lai *et al.* [80] have shown that maximin 1 and maximin 3 have microbicidal activities against both Gram positive and Gram negative bacteria and yeasts, *Candida albicans.* Both also have anti-cancer and anti-HIV activities. Although both maximin 1 and maximin 4 have no PDB ID, they are close homologs of maximin 4 (PDB: 2MHW). The 3D structure of maximin 4, as revealed by NMR analyses, is a linear cationic amphipathic peptide in solution, but forms a kinked alpha helix with lipid micelles [81].	[80]	The spermicidal work is based only on sperm immotility. At 100 μg/mL (37 μM) of maximin 1 or maximin 3, ~80% of human sperm become immotile.
Magainin 2 APD:AP00144 UniProt: P11006 for magainins; magainin 2 is one of the five cleaved products of magainins. PDB: 2MAG	Skin and stomach: African clawed frog, *Xenopus laevis*	Magainin 2 has microbicidal effects on Gram positive and Gram negative bacteria, yeasts and viruses. It also acts against certain protozoa including malaria causing *Plasmodium falciparum*. However, magainin 2 does not have an anti-HIV activity [82]. NMR analysis indicates that magainin 2 adopts an alpha helical structure in the presence of SDS and other lipid micelles [83].	[84,85,86,87]	Magainin A, a synthetic derivatives of magainin 2 [88] possess sperm immobilizing activity, as shown in rat, rabbit, monkey and human sperm. At ~50 μg/mL (20 μM), magainin A completely inhibit human/monkey sperm motility within 7–10 min of treatment, although it takes ~300 μg/mL (120 μM) of the peptide for the immediate immobilization of human sperm. Lower doses are required for rat and rabbit sperm for the same results [82,85,86]. These spermistatic effects are likely due to the ability of magainin A to disrupt the sperm surface membranes [85]. Magainin 2-amide (250 μg/mL (101 μM)) also exerts spermistatic effects on human sperm, although it is at 50% efficacy. Only when sperm are treated with both cyclodextrin and magainin 2-amide, sperm immotility is enhanced to 80%. Reddy’s group have demonstrated vaginal contraceptive effects of magainin A in rabbits and monkeys. Females each administered with 1 mg magainin A did not become pregnant upon natural mating. Side effects of magainin A on the female reproductive tract appeared to be minimum [82,84].
Dermaseptin S1 APD:AP00157 UniProt: 80277 PDB:None	Skin: Sauvage’s leaf frog, *Phyllomedusa sauvagii*	Dermaseptin S1 has microbicidal activities against Gram positive and Gram negative bacteria and herpes simplex virus. It also kills the protozoa, Leishmania. Dermaseptin S1 has an alpha helix structure as revealed by circular dichroism analyses [89].	[90]	The spermicidal work is based only on sperm immotility. Human sperm become completely immotile immediately after treatment with 200 μg/mL (58 μM) of dermaseptin S1.
Dermaseptin S4 APD:AP00160 UniProt: P80280 PDB: 2DD6 for a close analog of truncated dermaseptin-S4 (aa1-13).	Skin:Sauvage′s leaf frog, *Phyllomedusa sauvagii*	Dermaseptin S4 has microbicidal activities against Gram positive and Gram negative bacteria and viruses (herpes simplex virus and HIV). It also kills *P falciparum* protozoa. NMR analyses of a close analog of truncated dermaseptin S4 (aa1-13) indicates its alpha helix structure when interacting with lipid micelles [91].	[90,92,93]	Despite a similar structure to dermaseptin S1, only 100 μg/mL (36 μM) of dermaseptin S4 is required to induce complete human sperm motility, indicating a twice spermistatic potency of dermaseptin S4 [90]. A higher spermistatic effect is further observed in a dermaseptin S4 derivative with one amino acid replacement with lysine to increase the positive charge. Only 20 μg/mL (7.2 μM) of this derivative is required to induce complete sperm immotility effects. The native dermaseptin S4 (100 μg/mL (36 μM)) and its derivative (20 μg/mL (7.2 μM)) cause 100% and 50% cytotoxicity to HeLa cells, respectively [92].
Sarcotoxin Pd APD:AP02212 UniProt: None for this sarcotoxin but available for other sarcotoxin isoforms. PDB:None	Insects: rove beetles, *Paederus dermatitis*	Sarcotoxin Pd has microbicidal effects on Gram positive and Gram negative bacteria. Although there is no PDB information on sarcotoxin Pd, in the publication of Zare-Zardini *et al.* [94], a 3D structure of sarcotoxin Pd was shown to consist of two alpha helices. This structural information was obtained from computational modeling, although no details were given on how this modeling was performed.	[94]	The spermicidal work is based only on sperm immotility. The concentration of sarcotoxin Pd to immobilize human sperm is 80 μg/mL (22 μM). Cytotoxicity MTT assay was done on HeLa cells; at 80 μg/mL of sarcotoxin Pd, close to 100% of cells show cytotoxicity.
Nisin A APD:AP00205 UniProt:P13068 PDB: 1WCO	Bacteriocin from lactic acid bacteria (LAB), *Lactococcus lactis* (formerly called *Streptococcus lactis*)	The microbicidal effects of nisin A are more preferential to Gram positive bacteria; this may be attributed to its ability to bind to lipid II, a structural component of Gram positive bacterial peptidoglycans. The interaction between nisin A and lipid II leads to inhibition of the bacterial cell wall synthesis [95]. It is a cationic amphipathic lantipeptide (i.e., containing a lanthionine ether linkage between S3-C7 and 4 methyllanthionines: T8-C11, T13-C19, T23-C26 and T25-C28) (see Table 3). These thioether linkages create a constraint polycyclic feature to the peptide. However, NMR analysis revealed that nisin A is still flexible enough to interact with SDS micelles, first via ionic interaction and then through hydrophobic interaction. It was therefore proposed that nisin A can create pores in lipid bilayers following its tran-bilayer insertion [96,97].	[98,99,100,101,102,103]	The sperm immobilizing effects of nisin A have been shown in various species, *i.e.*, rats, rabbits, bulls, horses/ponies, boars, monkeys and humans [98,99,101]. The instantaneous spermistatic concentration of nisin A is 300–400 μg/mL (86–114 μM) for human/monkey sperm, 200 μg/mL (57 μM) for rabbit sperm and 50 μg/mL (14 μM) for rat sperm. Scanning electron microscopy revealed obvious disruption of the human sperm plasma membrane following treatment with 360 μg/mL (103 μM) of nisin A. This disruption was similar to what observed on the surface of *Staphylococcus aureus* bacteria, which were treated with a similar nisin A concentration. In contrast, human red blood cells were not affected by treatment with equivalent concentrations of nisin A. The preferential effects of nisin A on sperm plasma membrane permeabilization was also confirmed by propidium iodide nuclear staining of the treated sperm [102] Reddy *et al.* have further demonstrated that nisin A is an effective vaginal contraceptive in rats and rabbits. Female rats naturally cycling in the proestrous/estrous phase and vaginally administered with nisin A (200 μg each) did not become pregnant following natural mating [98]. Successful contraceptive results were likewise obtained in female rabbits intravaginally injected with nisin A (1 mg each), provided that mating took place within 30 min of the peptide administration [99]. In both animal species, the authors claimed that there were no changes to the anatomy of the female reproductive tract tissues or cytokine production profile in the females vaginally administered with nisin A at the contraceptive dose or even higher and repeated doses of nisin A [98,99,100]. By MTT assay, HeLa cells appeared to be less susceptible to cytotoxic effects of nisin A, as compared with sperm.
Pediocin CP2 APD:AP00634 UniProt: Q8RL96 PDB:None	Bacteriocin from LAB, *Pediooccus acidilactici*	Pediocin CP2 exerts microbicidal effects on both Gram positive and Gram negative bacteria as well as yeasts, *Candida albicans* [104] No PDB ID is available. However, the 3-D structure of its homolog, sakacin P (PDB: 1OG7, 68% identical peptide sequence to pediocin CP), reveals the presence of an alpha helix in the middle region of the peptide.	[104]	The spermicidal work is based only on sperm immotility. The concentration of pediocin CP2 to immobilize human sperm is >250 μg/mL (54 μM). There is no reported work on the cytoxicity concentration of pediocin CP2 on female reproductive tract tissues/cells.
Gramicidin A APD:AP00499 UniProt: None PDB: 1MAG	Bacteriocin from soil bacterium, *Bacillus brevis*	Gramicidin A exerts microbicidal effects on both Gram positive and Gram negative bacteria as well as viruses (including HIV) [105]. Gramicidin A has a special β-helix structure because of its possession of d-amino acids. Gramicidin A dimerizes with the N-terminus of each peptide being adjacent to each other. As a result, the dimer forms a cation channel in lipid bilayers with the two C-termini exposed [64].	[105,106,107]	Gramicidin has been used for a long time in Russia as a spermicide as referred to in Bourinbaiar *et al.* [105], although detailed data of its efficacy is not available. Experimentally, the spermicidal effects of gramicidin A are based on human sperm immotility and consequently the inability of sperm to penetrate lamb cervical mucus [106]. Only 5 μg/mL (2.8 μM) of gramicidin A could completely immobilize sperm. Gramicidin D (mixture of gramicidin A, B and C) has similar sperm immobilizing effects but at a higher concentration than gramicidin A.
Subtilosin A APD:AP00928 UniProt:007623 PDB: 1PXQ	Bacteriocin from *Bacillus subtilis*, *Bacillus amyloliquefaciens, Bacillus atrophaeus*	Subtilosin A has microbicidal effects on both Gram positive and Gram negative bacteria as well as herpes simplex viruses. It is a lantipeptide, containing a high percentage of hydrophobic residues (60%) with an overall negative charge of -2. Lanthionines between C13 and F22, C7 andT28, and C4 and F31 make the N-terminus and C-terminus fold towards each other, giving an overall boat conformation, as revealed by NMR analysis. There is an alpha helix in the C-terminus starting from G29 to W34 [108].	[101,109]	The spermicidal test is based only on sperm immotility. At a single concentration tested, 800 μg/mL (233 μM), subtilosin A instantaneously immobilized bull and horse/pony sperm. The same spermicidal effects were observed in boar and rat sperm at 200 μg/mL (58 μM) of subtilosin A [101]. Human sperm treated with subtilosin also became immotile in a dose-dependent manner with complete immobilization at 110 μg/mL (32 μM). Notably, subtilosin at this concentration does not reduce cell viability in the human EpiVaginal ectocervical tissue model [109].
Lacticin 3147 APD:AP01194 UniProt: 087236 PDB:None	Bacteriocin from LAB *Lactococcus lactis DPC3147*	Lacticin 3147 is composed of two lantipeptide components, LtnA1 and LtnA2. Microbicidal activity of lacticin 3147 is preferential on Gram positive bacteria and is stronger with LtnA1 and LtnA2 combined, compared with each lacticin 3147 chain alone. There are four lanthionines in LtnA1 and two in LtnA2, which generate a polycyclic structure to both peptides. NMR analyses also reveal a helical structure in LtnA2 [110]. Note that there are a few D-Ala residues in both lantipeptide chains, and their existence is important for the microbicidal activity of lacticin [110].	[101]	The spermicidal test is based only on sperm immotility. LtnA1 chain had much less spermicidal effects than LtnA2. The combination of LtnA1 + LtnA2 at 200 μg/mL (31 μM) could effectively immobilize rat, bull, and horse/pony sperm. But only 50 μg/mL (7.8 μM) of LtnA1 + LtnA2 was required to induce immotility of bull sperm [101].

^1^ The listing of spermicidal AMPs is essentially from the antimicrobial peptide database (APD) (http://aps.unmc.edu/AP/ [60]).

**Table 3:** Sequences and biochemical properites of spermicidal antimicrobial.

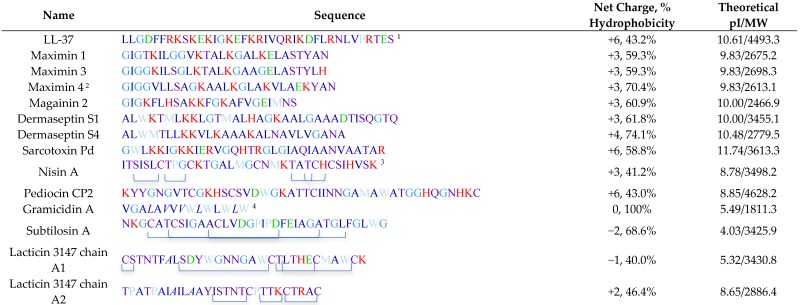

^1^ Amino acids are color coded according to their charges, hydrophilicity and hydrophobicity. Positively charged amino acids (K, R and H) are in red, whereas negatively charged residues (D, E) are in green. Uncharged hydrophilic amino acids (S, T, Y, N, Q, C) are in purple. Hydrophobic amino acids are in different shades of blue, with strong blue indicating the highest hydrophobicity level (L, F, I, V, A), followed by light blue (G), and very light blue (W, M, P) with the least hydrophobicity. ^2^ Although maximin 4 has not been shown to be spermicidal, its sequence is shown here because of its high similarity to maximin 1 and maximin 3 and its 3D structure is shown in Figure 3. ^3^ Brackets denote lanthionine ethers. ^4^ Italic letters denote d-amino acids.

**Table 4:** Microbicidal effects of LL-37.

Microbes ^1^	Bacteria Gram +/− (B+/−) Virus (V) Yeast (Y)	References: Concentration ^2^
Adenovirus (Ad)	V	Gordon *et al.* [160]: 111 μM
*Acinetobacter baumannii*	B−	Moffatt *et al.* [171]: 1.1 μM
		Garcia-Quintanilla *et al.* [172]: 0.67 μM
*Actinobacillus*	B−	Ouhara *et al.* [173]: ~0.26–0.52 μM
*Actinobacillus actinomycetemcomitans*	B−	Tanaka *et al.* [174]: ~2.2–2.7
*Actinobacillus actinomycetemcomitans*	B−	Ouhara *et al.* [173]: 2.2 μM
*Bacillus anthracis*	B−	Thwaite *et al.* [175]: 22 μM
*Borrelia burgdorferi*	Not applicable ^3^	Lusitani *et al.* [176]: 8.8 μM
*Borrelia spp*	Not applicable ^3^	Sambri *et al.* [169]: 100 μM
*Burkholderia pseudomallei*	B−	Kanthawong *et al.* [177]: 100 μM
*Burkholderia thailandensis*	B−	Kanthawong *et al.* [178]: 100 μM
*Candida albicans*	Y	Tsai *et al.* [179,180]: 8.9 μM
*Capnocytophaga gingivalis*	B−	Tanaka *et al.* [174]: 2.0 μM
*Capnocytophaga ochracea*	B−	Tanaka *et al.* [174]: 2.4 μM
*Chlamydia trachomatis*	B−	Tang *et al.* [181]: 20 μM
*Clostridium difficile*	B+	McQuade *et al.* [182]: 10.7 μM
*Enterococcus faecalis*	B+	Leszczynska *et al.* [183]: 12.5 μM
*Escherichia coli*	B−	Benincasa *et al.* [184]: 5 μM
		Smeianov *et al.* [168]: 25 μM
		Leszczynska *et al.* [185]: 5.6 μM
		Chen *et al.* [186]: 0.07 μM
		Kai-Larsen *et al.* [187]: 20 μM
		Nagaoka *et al.* [188]: ~1-2 μM
*Fusobacterium nucleatum*	B−	Ouhara *et al.* [173]: 0.22 μM
		Leszczynska *et al.* [183]: 49.8 μM
*Haemophilus influenzae*	B−	Leszczynska *et al.* [183]: 12.5 μM
		Lysenko *et al.* [189]: 2.2 μM
*Helicobacter pylori*	B−	Leszczynska *et al.* [183]: 6.2 μM
		Leszczynska *et al.* [185]: 2.2 μM
Herpes simplex virus type 1	V	Gordon *et al.* [160]: 111 μM
HIV-1	V	Wang *et al.* [135]: 1.6 μM
		Bergman *et al.* [134]: 11.1 μM
Influenza A virus (IAV)	V	Tripathi *et al.* [190]: 13 μM
		Barlow *et al.* [191]: 11.1 μM
		Tripathi *et al.* [192]: 6.7 μM
*Klebsiella pneumoniae*	B−	De Majumdar *et al.* [193]: >11.1 μM
*Moraxella catarrhalis*	B−	Leszczynska *et al.* [183]: 6.2 μM
*Neisseria gonorrhoeae*	B−	Bergman *et al.* [194]: 0.8 μM
*Neisseria meningitidis*	B−	Leszczynska *et al.* [183]: 12.5 μM for strain B
		Leszczynska *et al.* [183]: 24.9 μM for strain C
		Jones *et al.* [195]: 10 μM
*Peptostreptococcus anaerobius*	B+	Leszczynska *et al.* [183]: 49.8 μM
*Porphyromonas gingivalis*	B−	Leszczynska *et al.* [183]: 49.8 μM
		Ouhara *et al.* [173]: 11.1 μM
*Prevotella intermedia*	B−	Ouhara *et al.* [173]: 1.1 μM
*Pseudomonas aeruginosa*	B−	Bergsson *et al.* [196]: 5.6 μM
		Dean *et al.* [197]: 0.22 μM
		Dosler and Karaaslan [198]: ~14.2–28.4 μM
		Gordon *et al.* [160]: ~11.1–22.2 μM
		Leszczynska *et al.* [183]: 99.7 μM
Respiratory syncytial virus	V	Currie *et al.* [199]: 5.6 μM
*Staphylococcus aureus*	B+	Leszczynska *et al.* [183]: 6.2 μM
		Noore *et al.* [200]: 2 μM
		Chen *et al.* [186]: 0.67 μM
		Senyurek *et al.* [201]: 11.1 μM
		Nagaoka *et al.* [188]: 1 μM
		Gordon *et al.* [160]: ~11.1–22.2 μM
*Staphylococcus epidermidis*	B+	Leszczynska *et al.* [183]: 12.5 μM
		Gordon *et al.* [160]: ~11.1–22.2 μM
*Streptococcus mitis*	B+	Ouhara *et al.* [173]: 2.2 μM
*Streptococcus mutans*	B+	Ouhara *et al.* [173]: 0.22 μM
		Leszczynska *et al.* [183]: 6.2 μM
*Streptococcus pneumoniae*	B+	Nagaoka *et al.* [188]: 1 μM
		Leszczynska *et al.* [183]: 3.1 μM
*Streptococcus pyogenes*	B+	Leszczynska *et al.* [183]: 3.1 μM
*Streptococcus salivarius*	B+	Ouhara *et al.* [173]: 1.1 μM
		Leszczynska *et al.* [183]: 6.2 μM
*Streptococcus sanguis*	B+	Ouhara *et al.* [173]: 0.22 μM
		Leszczynska *et al.* [183]: 6.2 μM
*Streptococcus sobrinus*	B+	Ouhara *et al.* [173]: 1.1 μM
*Tannerella forsythensis*	B+	Leszczynska *et al.* [183]: 49.8 μM
*Treponema pallidum*	B-	Sambri *et al.* [169]: 100.1 μM
*Ureaplasma parvum*	NA ^3^	Xiao *et al.* [202]: 22.2 μM
*Ureaplasma urealyticum*	NA ^3^	Xiao *et al.* [202]: 22.2 μM
Vaccinia virus	V	Howell *et al.* [203]: 20 μM
Varicella zoster virus (VZV)	V	Crack *et al.* [204]: 0.1 μM

^1^ Highlighted microbes: blue causing STI; green causing vaginitis; Red causing UTI; Pink causing vaginitis and UTI. **^2^** Concentrations of LL-37 given are those that exert microbicidal effects on ≥90% of the microbes. In some cases where this information is not clearly described in the publication, estimated concentrations are given. ^3^ NA = not applicable; *Borrelia* spp. and *Ureaplasma* spp. do not react well with the Gram stain.
